# Arenobufagin-loaded PEG-PLA nanoparticles for reducing toxicity and enhancing cancer therapy

**DOI:** 10.1080/10717544.2023.2177362

**Published:** 2023-02-11

**Authors:** Yang Jiaying, Sun Bo, Wei Xiaolu, Zhou Yanyan, Wang Hongjie, Si Nan, Gao Bo, Wang Linna, Zhang Yan, Gao Wenya, Luo Keke, Jiang Shan, Luo Chuan, Zhao Yu, Zhao Qinghe, Zhao Haiyu

**Affiliations:** a China Academy of Chinese Medical Sciences, Institute of Chinese Materia Medica, Beijing, China; b China Resources Sanjiu Modern Traditional Chinese Medicine Pharmaceutical Co., Ltd, Shenzhen, China; c Anhui Huarun Jinchan Pharmaceutical Co., Ltd, Anhui, China

**Keywords:** Arenobufagin, polymeric micelles, pharmacodynamics, drug delivery, cancer

## Abstract

Arenobufagin (ArBu) is a natural anticancer drug with good anti-tumor effects, but its clinical applications and drug development potential are limited due to its toxicity. The purpose of this study is to reduce the toxic side effects of ArBu and improve the efficacy of tumor treatment by incorporating it into poly(ethylene glycol)-b-poly (lactide) co-polymer (PEG-PLA). ArBu@PEG-PLA micelles were prepared by a thin film hydration method. The optimized micelles were characterized by size, stability, drug loading, encapsulation rate, and drug release. The tumor-inhibition efficacy of the micelles was evaluated on A549 cells and tumor-bearing mice. The ArBu@PEG-PLA micelles have good drug-loading capacity, release performance, and stability. They can accumulate at the tumor site through the EPR effect. The micelles induce apoptosis through a mitochondrial apoptosis pathway. Compared with the free ArBu, the ArBu@PEG-PLA micelles had lower toxicity and higher safety in the acute toxicity evaluation experiment. The *in vivo* anti-tumor experiment with tumor-bearing mice showed that the tumor-inhibition rate of ArBu@PEG-PLA micelles was 72.9%, which was 1.28-fold higher than that of free ArBu (57.1%), thus showing a good tumor treatment effect. This study indicates that ArBu@PEG-PLA polymeric micelles can significantly improve the toxicity and therapeutic efficacy of ArBu. These can lead to a new therapeutic strategy to reduce the toxicity of ArBu and enhance tumor treatment.

## 1. Introduction

Anticancer drugs from natural sources have attracted a great deal of attention worldwide (Wang & Yuan, 2013; Li & Leung, 2014; Xia et al., 2017; Liu et al., 2019; Wang et al., 2019; Hao et al., 2020; Luo et al., 2021).

Arenobufagin (ArBu) is the major pharmacological constituent of the traditional Chinese medicine Chan’su and several well-known Chinese patent medicines such as Toad Venom Injection and Huachansu Injection, which are used in chemotherapy either alone or in combination for cancer treatment in China (Yu et al., 2014). Currently, several experimental and clinical studies have suggested that ArBu has significant antitumor activity toward breast cancer, non-small-cell lung cancer, liver cancer, and gastric cancer (Zhang et al., 1992; Chen et al., 2003; Meng et al., 2009; Zhang et al., 2013; Deng et al., 2015). However, ArBu is an Na^+^/K^+^-ATPase inhibitor and exhibits significant cardiotoxicity such as fatal arrhythmia and cardiac fibrosis (Kostakis & Byard, 2009). The pharmacokinetic characteristics of ArBu include poor stability, short half-life (T_1/2_), low bioavailability, low water-solubility and low blood concentration. These features limit its clinical usage and potential for drug development (Xie et al., 2001; Kostakis & Byard, 2009; Ying et al., 2011; Li et al., 2013, 2015; Arnold & Das, 2018). ArBu has recently been modified by chemical synthesis or biological transformation. The toxicity of ArBu analogues has been evaluated in vitro. Unfortunately, these efforts have shown little evidence of reduced toxicity. Consequently, new approaches are needed to extend these studies toward reducing or relieving the toxicity, enhancing the efficacy, and improving the biopharmaceutical effects of ArBu when used as an anticancer agent.

Nano-drug delivery systems are an important tool to improve drug solubility and tumor targeting and to reduce drug toxicity. Polymer-based nanoparticles show unique advantages in terms of their simple preparation, high stability, and flexible adjustment. Polymeric micelles (Kim et al., 2001, 2005; Lee et al., 2008; Cabral & Kataoka, 2014), are a promising drug-delivery nanovector that can accumulate in the tumor through the well-known ‘EPR effect’ (Maeda et al., 2000; Torchilin, 2011), due to the abnormal culture and lymphatic vessels. They have attracted increasing attention for treating cancer (Fang et al., 2020). Yuan et al. designed ArBu-loaded nanoparticles for cancer therapy (Yuan et al., 2017): these micelles showed increased concentrations of ArBu in the lungs and liver. In contrast, the concentrations of the drug in both the heart and brain were reduced. ArBu-loaded nanoparticles may enhance the anticancer effects of free ArBu by increasing the cellular uptake.

Polyethylene glycol (PEG) is a hydrophilic polymer with good biocompatibility and water solubility, and it can effectively avoid clearance from the blood circulation. Poly-lactide (PLA) has been used as a hydrophobic segment in polymeric micelles for drug delivery systems. PLA is usually compatible with low water-solubility drugs, and it has good biodegradability (Kim et al., 2017). Both are approved by the Food and Drug Administration (FDA) for use in food, medicine, and more (Tipnis & Burgess, 2018; Park et al., 2019). The Poly (ethylene glycol)-b-poly(lactide) (PEG-PLA) micelles have superior penetration abilities in solid tumors and can deliver various chemotherapeutic agents to sites of metastasis after systemic injection, thus promoting their efficacy in suppressing a variety of cancer types.

**Scheme 1.**
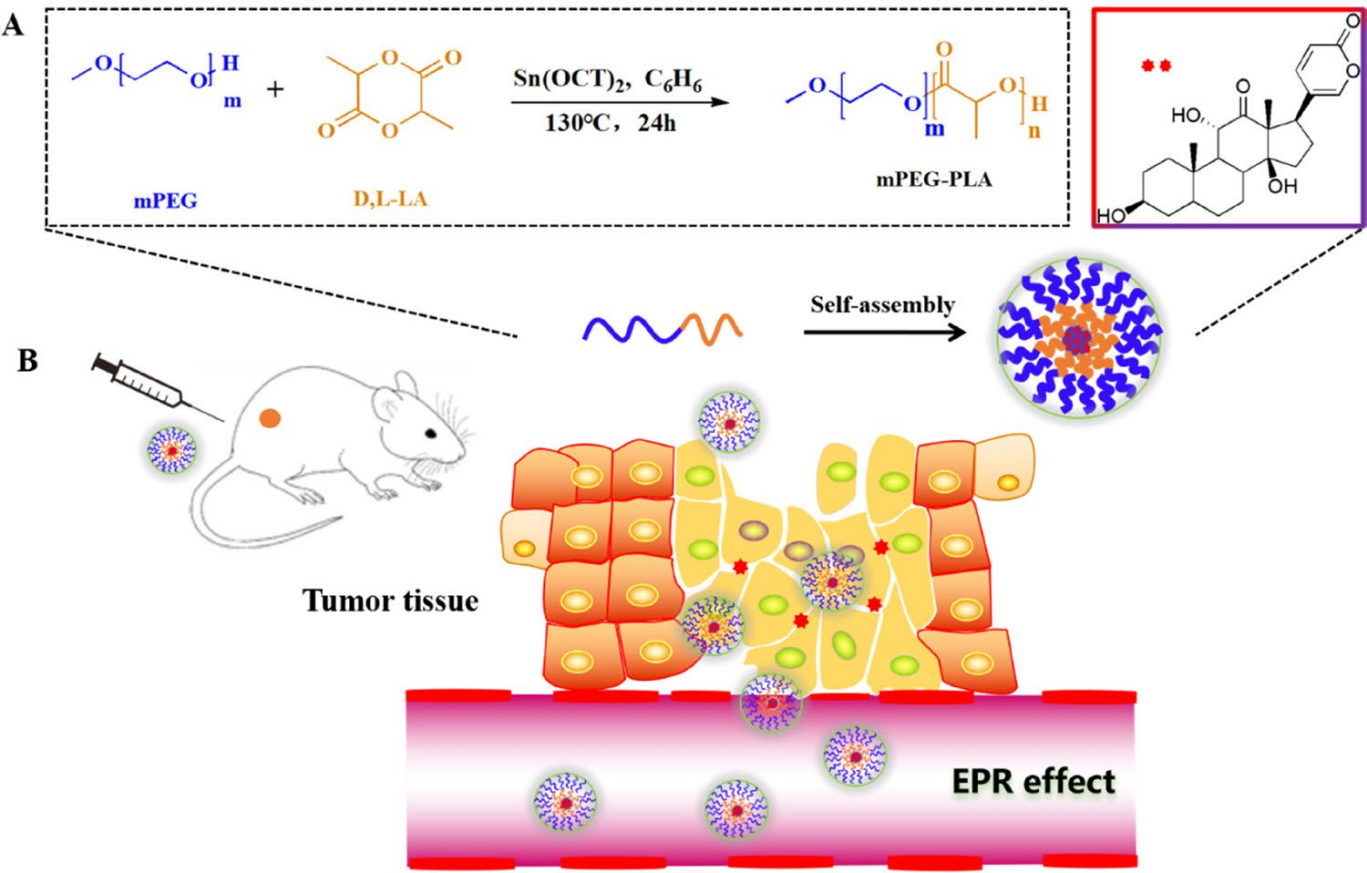
(A) Schematic illustration of the fabrication process of ArBu@PEG-PLA micelles. (B) The ArBu@PEG-PLA micelles mediated antitumor synergistic therapy by EPR effect.

Here, we proposed an Arbu-loaded polymeric micelle (ArBu@PEG-PLA) nanoplatform to achieve accumulation of the anticancer drug ArBu in tumor sites via the EPR effect (Scheme 1). The goal is to improve the anticancer and safety properties of the drug and thus increase the therapeutic window of ArBu. The ArBu@PEG-PLA micelles were combined with the active ingredient in the traditional Chinese medicine Chan’su to improve the ArBu utilization rate. The Arbu could be encapsulated in the PEG-PLA micelles to enhance its solubility. PEG was used as the hydrophilic part of ArBu@PEG-PLA to increase the circulation time *in vivo* and to accumulate the nanoparticles at the tumor site through the EPR effect. This in turn offers passive targeting of the tumor site while reducing the drug concentrations in normal tissues (thus reducing toxicity). The ArBu was then gradually released from ArBu@PEG-PLA micelles upon degradation of PLA. This led to slow release in the tumor for long-term treatment. The outcomes have significant benefit for preclinical development and clinical applications of ArBu as a cancer drug. Thus, the ArBu@PEG-PLA micelles are promising for increasing the value of ArBu.

## 2. Materials and methods

### 2.1. Materials

Methoxy PEG (MPEG, Mw2000), tin (II)2-ethylhexanoate, 4′,6-dia-midino-2-phenylindole (DAPI) and coumarin-6 (C6) were purchased from Sigma-Aldrich (St. Louis, MO, USA). D,L-lactide and polyethylene glycol were purchased from TCI (Tokyo, Japan n), and 3-(4,5-dimethylthiazol-2-yl)-2,5-diphenyltetrazolium bromide (MTT) was obtained from Beyotime (Shanghai, China). Fetal bovine serum (FBS), McCoy’s 5 A medium, and trypsin-EDTA were purchased from Gibco Company (San Diego, CA, USA). All other chemicals were of analytical or HPLC grade and were used as received. A549 cells were purchased from the National Infrastructure of Cell Line Resource (Beijing, China) and cultured with McCoy’s 5 A containing 10% FBS and 1% penicillin-streptomycin in an incubator with 5% CO_2_ at 37 °C.

### 2.2. Preparation of PEG-PLA

PEG-PLA was synthesized by ring opening polymerization. Briefly, D, L-lactide and PEG were dissolved at a mass ratio of 1:1 in 20 mL anhydrous toluene and initiated by tin (II)2-ethylhexanoate as a catalyst at 130 °C for 24 h. The PEG-PLA polymer micelles were obtained by precipitation in diethyl ether and vacuum drying (Chen et al., 2017).

### 2.3. Preparation of ArBu@PEG-PLA

The ArBu@PEG-PLA was synthesized by thin-film hydration (Chen et al., 2017). Briefly, PEG-PLA co-polymer and ArBu with a predetermined weight ratio were dissolved in 5 mL MeOH. The solvent was then vaporized at 50 °C to form a solid film. The film was dispersed by deionized water, and the solution was filtered through a 0.22 μm filter (Esselink et al., 1993). The preparation method of C6-loaded PEG-PLA polymer micelles was the same as above. The ArBu@PEG-PLA micelles were evaluated by transmission electron microscopy (TEM) (Zhang et al., 2020). Samples for TEM were prepared by re-dispersing in water and then casting them onto TEM grids. The size of the micelles was measured using a Malvern Zetasizer Nano ZS90 (Worcestershire, United Kingdom) with a detection angle of 90°. After preparation of the samples, the particle sizes were measured within 7 days to characterize the micelle stability. The amount of loaded ArBu was determined by ultra-performance liquid chromatography (UPLC). The loading capacity (LC) and encapsulation efficiency (EE) were calculated according to the following equations (Jia et al., 2021):

$$\mathrm{LC}\;(\%)\;=\frac{\text{weight}\;\text{of}\;\text{drug}\;\text{loaded}\;\text{micelles}}{\text{weight}\;\text{of}\;\text{drug}\;\text{in}\;\text{the}\;\text{micelles}}\;*100\%$$

$$\mathrm{EE}\;(\%)\;=\frac{\text{weight}\;\text{of}\;\text{drug}\;\text{in}\;\text{the}\;\text{micelles}}{\text{weight}\;\text{of}\;\text{the}\;\text{added}\;\text{drug}}\;*100\%$$

### 2.4. In vitro drug release

The release behavior of ArBu@PEG-PLA micelles in vitro was investigated by dialysis. First, 3 mL of ArBu@PEG-PLA micelles was encapsulated in dialysis bags (MWCO 3500) and then immersed in 20 mL PBS (pH 7.4) at 37 °C. The original dialysis medium was replaced and an equal volume of fresh dialysis medium was added at different time points (0.125, 0.25, 0.5, 1, 2, 4, 8, 12, 24, and 48 h) (Hao et al., 2022). The release amount of ArBu in micelles was detected by UPLC. The mobile phase consisted of acetonitrile (A) and water (B). A gradient program was used as follows: 0–4 min, 30–62% A; 4–5 min, 62–95% A; and 5–10 min, 95–100% A. The flow rate was set at 0.3 mL/min, and the injection volume was 5 μL. The column temperature was 26 °C, and the detection wavelength was 299 nm.

### 2.5. Cellular uptake

Cellular uptake of free C6 and C6-loaded PEG-PLA micelles was evaluated using confocal laser scanning microscopy (CLSM) and flow cytometry (Yang et al., 2020). The CLSM observations used A549 cells seeded in 12-well plates at 2 × 10^5^ cells per well. After 12 h of incubation, the A549 cells were treated with C6 and C6-loaded micelles (C6@PEG-PLA, micelle concentration: 50 μg/mL) in McCoy’s 5 A medium with 10% FBS. The A549 cells were then washed three times with 4 °C PBS and then fixed with 4% paraformaldehyde for 15 min. DAPI solution was then added to the A549 cells for 10 min. Finally, the cells were observed using CLSM after washing with cold PBS. In the flow cytometry experiments, the cells were treated similarly as in the CLSM. They were finally resuspended in PBS for detection. The suspended A549 cells were directly analyzed with BD flow cytometry (FACS Calibur, USA).

### 2.6. In vitro cytotoxicity and cell apoptosis analysis

The cytotoxicity of micelles was evaluated with MTT assay (*n* = 6). The cells were seeded in a 96-well plate at 1 × 10^4^ cells per well for 24 h. Next, the cells were treated with various concentrations of ArBu@PEG-PLA micelles. The cell viability was measured via a microplate reader at 570 nm after 24 h of incubation (Huang et al., 2022).

For cell apoptosis assay, the cells were seeded in a 12-well plate at 2 × 10^5^ cells per well. After 24 h of incubation, various concentrations of ArBu@PEG-PLA (ArBu dosage 100 μM) were added to the cell media and incubated for 24 h. The cells were then washed three times with cold PBS and stained with Annexin V-FITC and propidium iodide (PI) for 15 min in the dark. The treated cells were then resuspended in PBS and evaluated with flow cytometry (Xiao et al., 2021).

### 2.7. Live/dead cell staining assay

A549 cells were seeded in 6-well plates and incubated for 24 h. The cells were incubated with ArBu or ArBu@PEG-PLA (ArBu dosage 100 μM) for 12 h. All cells were then stained with calcein acetoxymethyl ester (Calcein-AM, 2 μM) and PI (4 μM) at 37 °C for 30 min. Finally, the cells were washed with cold PBS three times and visualized using CLSM (Fu et al., 2021).

### 2.8. Mitochondrial membrane potential assay

Mitochondrial membrane potential was measured using the JC-1 Assay Kit in PBS and then measured with flow cytometry (Dong et al., 2020). The cells were first seeded in 96-well plates and incubated for 24 h. The cells were then treated with ArBu and ArBu@PEG-PLA (ArBu dosage: 100 μM) and then incubated for 24 h. The change in mitochondrial membrane potential was measured using fluorescence incubation Probe JC-1 at 37 °C for 15 min. The cells were analyzed by flow cytometry after rinsing with JC-1 incubation buffer three times.

### 2.9. Western blot analysis

A549 cells were seeded in 6-well plates at 1 × 10^5^ cells per well, cultured for 12 h, and then incubated with free ArBu and ArBu@PEG-PLA (ArBu dosage: 100 μM) for 24 h. After being washed with PBS, the cells were incubated with lysis buffer containing 1 mmol phenylmethylsulfonyl fluoride for 30 min. The protein content was determined by the Bradford method. Western blot analysis was performed using standard methods, and GAPDH was used as the internal reference (Ding et al., 2022). The intensities of the proteins were determined using Image J software.

### 2.10. Acute toxicity assay

Here, 100 Kunming mice (22.0 − 28.0 g; both males and females) were randomly divided into ArBu and ArBu@PEG-PLA groups. Intraperitoneal injection with ArBu and micelles (6, 10, 15, 19, and 24 mg/kg, *n* = 10) was performed on day 1. The Kunming mice were then observed continuously for 10 h after drug administration. Abnormal behavior and death rate were recorded. The mice were then observed for the next 7 days. SPSS was used to calculate the LD50 values of ArBu and ArBu@PEG-PLA micelles (Deng et al., 2017).

### 2.11. Animals and tumor xenograft models

The female Balb/c nude mice (6–8 weeks old) weighed 19–23 g. To establish the tumor model, 6 × 10^6^ A549 cells were subcutaneously injected into the right lower limb of the mice. All animal studies followed the Guidelines for the Care and Use of Laboratory Animals. The tumor volume was measured every 3 days. This was then calculated as follows: V = (length) × (width)^2^/2 (Zhang et al., 2022).

### 2.12. In vivo antitumor effect

When the volume of tumor reached about 100 mm^3^, A549 tumor-bearing mice were randomly divided into three groups (*n* = 3) (Yu et al., 2019): PBS group (control group), ArBu group, and ArBu@PEG-PLA group. The tumor-bearing mice were intraperitoneally injected with free ArBu or ArBu@PEG-PLA (ArBu: 5 mg/kg^−1^, micelles: 5 mg/kg^−1^). The tumor volumes were monitored every 2 days for a total of 10 days. Mice were killed on the 21^st^ day. The tumor tissues and the major organs (heart and kidney) were excised and weighed and prepared for H&E staining (Yu et al., 2022).

### 2.13. Statistical analysis

In this article, all data were processed using GraphPad Prism 8 software and presented as mean ± standard deviation (SD). The statistical analysis was performed by using one-way analysis of variance (ANOVA). The difference was indicated statistically significant as *p < .05, **p < .01, and ***p < .001 (Geng et al., 2021).

## 3. Results and discussion

### 3.1. Preparation and characterization of PEG-PLA

**Figure 1.**
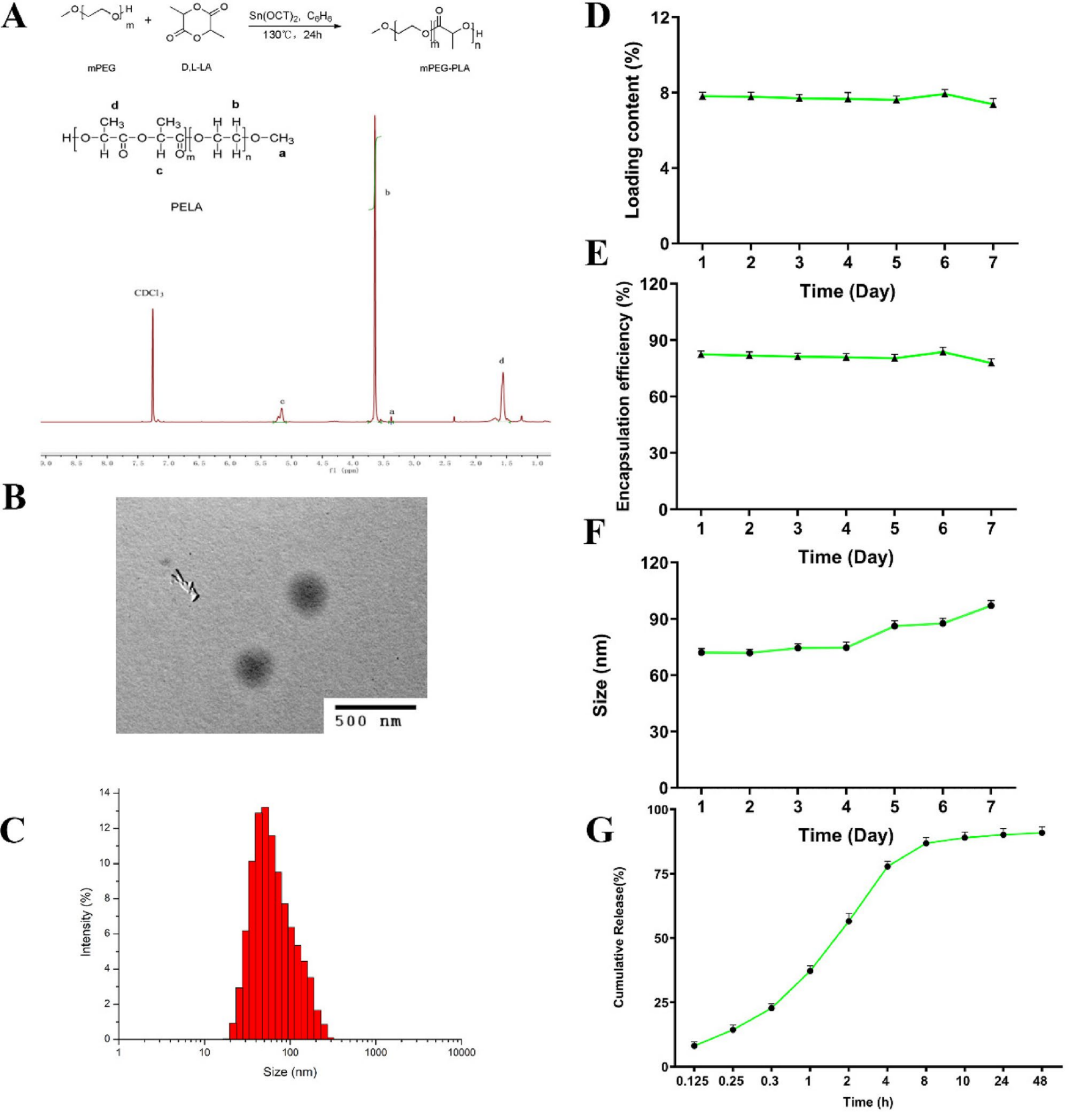
(A) ^1^H-NMR spectra of PEG-PLA micelles (CDCl_3_, 600 MHz). (B) TEM results of ArBu@PEG-PLA micelles. (C) DLS size of ArBu@PEG-PLA micelles. (D–F) Stability test results of loading content, encapsulation efficiency and size of ArBu@PEG-PLA micelles at 37 °C within 7 days, respectively (*n* = 3). (G) In vitro release of ArBu from ArBu@PEG-PLA micelles in PBS at pH 7.4.

PEG-PLA was synthesized by ring-opening polymerization (Qiu et al., 2013; Chen et al., 2017). The chemical structure of PEG-PLA was characterized and confirmed by ^1^H NMR spectroscopy (Figure 1A). The proton shift peaks included δ3.38 (PEG, -OCH_3_), 3.64 (PEG, -OCH_2_CH_2_-), 1.56 (PLA, -CH_3_) and 5.16 (PLA, -CO-CH(CH_3_)-O-), δ 5.91–6.19 (acryloyl, -O-CO-CH=), and 6.47 (acryloyl, =CH_2_). These peaks confirmed the successful synthesis of PEG-PLA. Using ^1^H NMR, the average molecular weight (M_n_) of the co-polymer was found to be 4008 based on the integral height ratio of hydrogen on different target groups in the nuclear magnetic spectrum.

### 3.2. Preparation & characterization of ArBu-loaded micelles

ArBu-loaded PEG-PLA micelles (ArBu@PEG-PLA) were synthesized via the thin film hydration method. The morphology of ArBu@PEG-PLA micelles was characterized by TEM, and the results showed that the micelles were spherical (Figure 1B). The ArBu-loaded PEG-PLA co-polymer micelles had small sizes and a narrow distribution.

The size of ArBu@PEG-PLA micelles in aqueous solution was determined by dynamic light scattering (DLS). The size of the polymer micelles was 72–97 nm with a PDI of 0.104 (Figure 1C). This may promote the accumulation of the polymer in tumor tissues. The sizes of the ArBu@PEG-PLA micelles increased significantly compared to those of PEG-PLA co-polymers. This indicates that the molecular structure of the polymer is more rigid due to the introduction of hydrophobic ArBu molecules in the core of the amphiphilic co-polymer (Kang et al., 2017). All micelles showed high drug loading content (7.3–7.5%) (Figure 1D) and drug encapsulation efficiency (80.3–82.5%) (Figure 1E). The stability of the ArBu@PEG-PLA micelles was investigated by measuring the micelles’ size changes at 37 °C. At room temperature, all ArBu@PEG-PLA micelles remained stable in terms of size (71–74 nm with a PDI of 0.114 within 5 days; determined by monitoring the transmittance). From 5 to 7 days, the size of ArBu@PEG-PLA micelles increased dramatically (86–97 nm), with a PDI increase to 0.121. The results showed that the structure of the ArBu@PEG-PLA micelles remained stable for 5 days. They gradually increased beyond 5 days (Figure 1F).

### 3.3. In vitro drug release

The in vitro drug release of ArBu@PEG-PLA micelles were performed at pH 7.4 (Gong et al., 2012). Figure 1(G) shows that the ArBu in the micelles was gradually released in a stable and durable way upon degradation of PLA. This approached a maximum of 90.9% of ArBu release within approximately 48 h at pH 7.4. The drug was released slowly upon gradual degradation of the hydrophobic PLA segment (Bohr et al., 2017; Chen et al., 2021). The degradation rate of PLA was related to its molecular weight. The rate of polymer degradation decreases with increasing molecular weight, thus slowing the rate of drug release. There was no initial burst release. These results showed that ArBu@PEG-PLA micelles had good release performance under normal physiological conditions. The long-term in vivo circulation characteristics of PEG increase the concentration of ArBu at the tumor site.

### 3.4. In vitro cellular uptake

**Figure 2.**
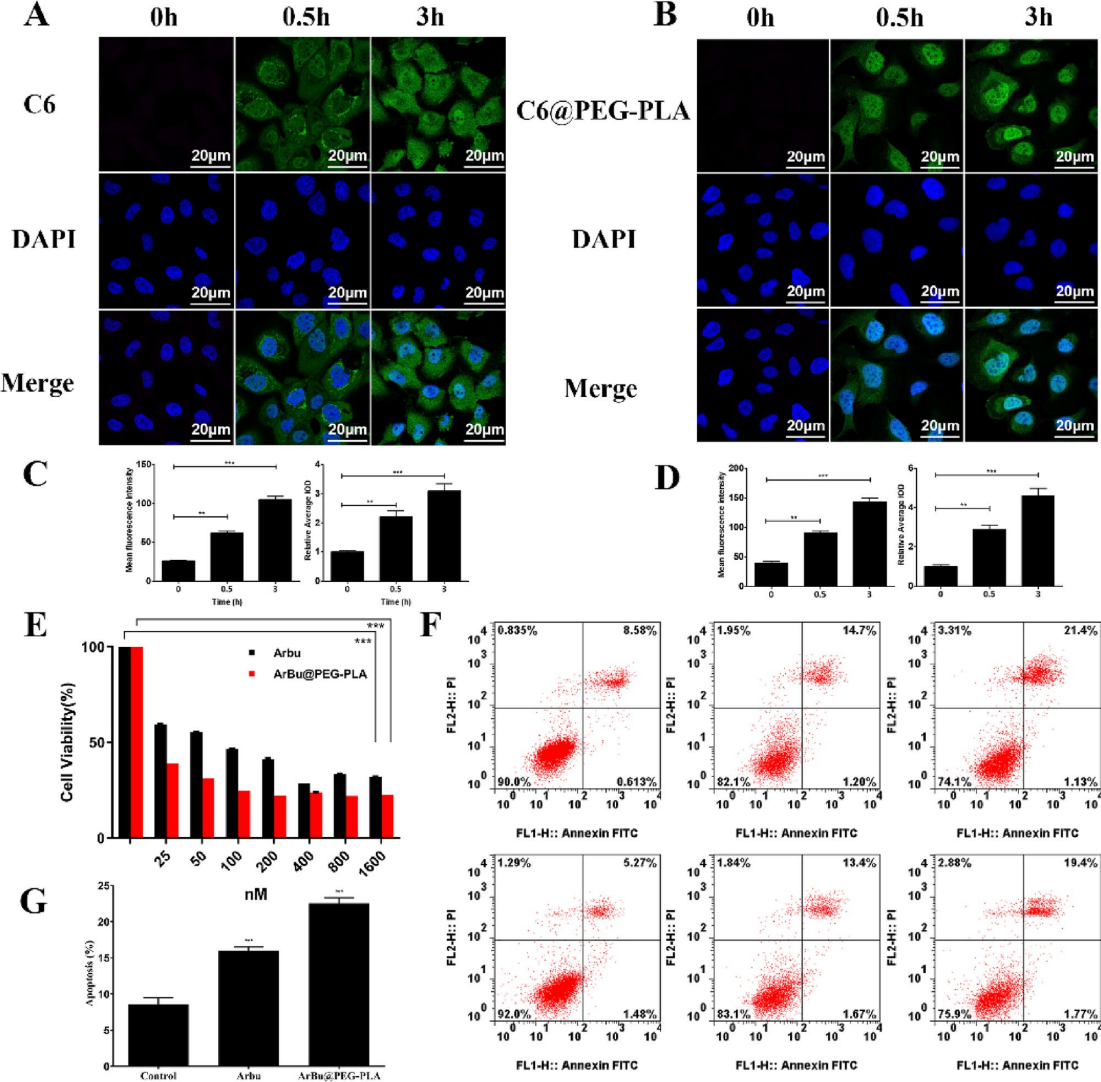
(A, B) Confocal FL images of cellular internalized C6 and C6@PEG-PLA, respectively, after 3h incubation with A459 cells. Blue FL represents DAPI, green FL represents C6. (C, D) Flow cytometry analysis of mean FL intensity (*n* = 10,000 cells) in A459 cells incubated with C6 and C6@PEG-PLA for 0.5 h and 3h, respectively. Data are presented as the mean of three measurements ± SD. *p < .05, **p < .01, ***p < .001. (E) Cell viability of A459 cells after treated free ArBu and ArBu@PEG-PLA micelles. (F) The apoptosis rates of A459 cells after incubation with free ArBu and ArBu@PEG-PLA micelles. Flow cytometry analysis via Annexin V/PI staining. (G) Quantitative analysis of tumor cells apoptosis after incubation with free ArBu and ArBu@PEG-PLA micelles for 24 h. *p < .05, **p < .01, ***p < .001.

Coumarin-6 (C6) was incorporated into PEG-PLA micelles as a model drug to investigate the cellular internalization of the micelles. CLSM was used to observe the cellular internalization of C6-incorporated micelles. Figure 2(A)–(D) shows that only a weak fluorescence was observed in the cytoplasm after 4 h of incubation with free C6. Cells incubated with C6@PEG-PLA micelles showed stronger fluorescence in the cytoplasm. The flow cytometry analysis also showed a significant improvement in the fluorescence in the C6@PEG-PLA micelles group, which matched the experimental results of CLSM. These results collectively indicated that small micelles could more easily enter the A549 cells, thus enhancing accumulation at the tumor site.

### 3.5. Cytotoxicity assay and cell apoptosis analysis

We used the methyl thiazolyl tetrazolium (MTT) assay to evaluate the in vitro antiproliferative activity of ArBu@PEG-PLA micelles. We first studied the cytotoxicity of PEG-PLA in A549 cells. There was no obvious cytotoxicity with PEG-PLA (Figure 2E). Free ArBu exhibits cell-killing activity in A549 cells. The lowest half maximal inhibitory concentration (IC50) was 71 nM. In contrast, ArBu@PEG-PLA micelles exhibited increased efficacy compared to free ArBu (IC50 of 11 nM in A549 cells).

We also studied the cellular apoptosis in free ArBu and ArBu@PEG-PLA micelles via Annexin V-FITC/PI apoptosis detection (Figure 2F and 2G). The apoptosis rates of cells incubated with free ArBu and ArBu@PEG-PLA micelles for 24 h were 18.6% and 35.1%, respectively. This result is consistent with the MTT analysis results. The results again confirmed that the ArBu@PEG-PLA micelles could enhance the intracellular accumulation of ArBu and kill more cancer cells. In turn, the G2/M arrest of A549 cells treated with ArBu@PEG-PLA micelles was significantly higher than that of A549 cells treated with free ArBu, which proved that micelles were effective in helping drugs enter cells. The micelles can deliver more drugs into the cell, and thus, apoptosis was more significant in the G2/M phase.

### 3.6. Live/dead cell staining assay

**Figure 3.**
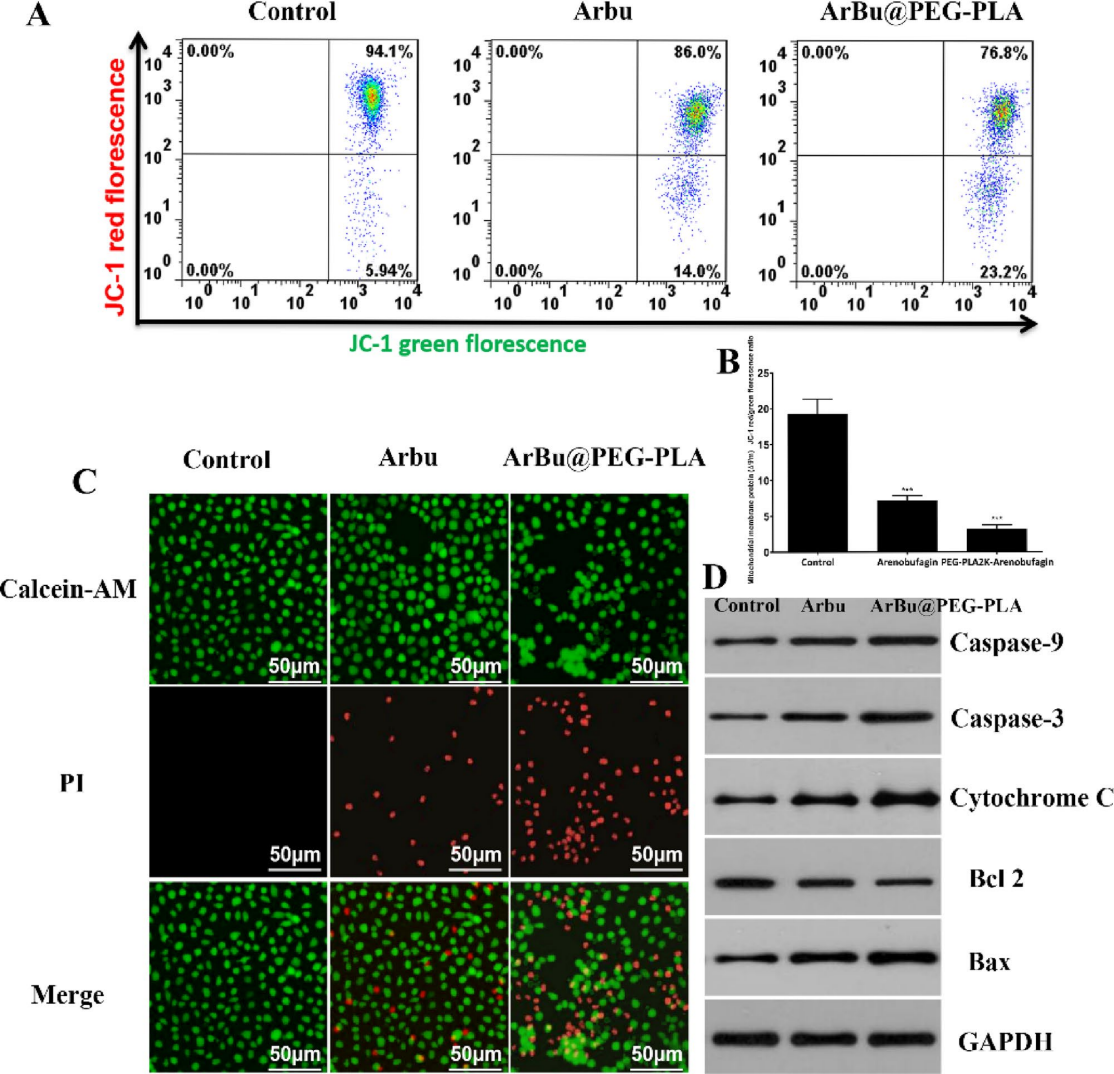
(A) Mitochondrial membrane potential changes of A459 cells after different treatments. (B) Relative intensities of the expression of mitochondrial membrane potential after incubation of A459 cells with free ArBu and ArBu@PEG-PLA micelles for 24h. (C) Live/dead assay, fluorescence images of AM (green) and PI (red) co-staining A459 cells incubated with free ArBu and ArBu@PEG-PLA micelles. Scale bar, 50 μm. (D) Western blotting analyses of A459 cells after incubation with free ArBu and ArBu@PEG-PLA micelles for 24 h, GAPDH as control.

We next visualized the distribution of living and dead cells: A549 cells were treated with ArBu and ArBu@PEG-PLA (ArBu dosage: 100 μM) for 12 h and then cultured with calcein-AM (2 μM) and PI solutions (4 μM). The live cells were stained green, and dead cells were stained red. Figure 3(C) shows that the cells in the control group without any treatment grew well, but a minor inhibition was observed for the cells treated with free ArBu. Importantly, most cells that were treated with ArBu@PEG-PLA died. These results proved that ArBu@PEG-PLA micelles could effectively improve cell killing, and thus, they have a significant anti-tumor effect.

### 3.7. Mitochondrial membrane potentials

Apoptosis is closely related to mitochondria dysfunction. To further investigate the possible mechanism involved in increased cytotoxicity, we next used JC-1 staining to observe the changes of mitochondrial membrane potential. Figure 3(A) and 3(B) shows the comparison of the free ArBu group with the ArBu@PEG-PLA micelle group. The latter showed a higher fluorescence ratio of green to red for 24 h, suggesting that numerous mitochondria were damaged. These results suggested that ArBu@PEG-PLA micelles have obvious mitochondrial apoptosis. The breakdown of the mitochondrial membrane leads to the death of tumor cells, thus showing better anti-tumor effects.

### 3.8. Western blot analysis

We next further confirmed the relationship between apoptosis and mitochondrial pathway: cell apoptosis was investigated by Western blot analysis after incubating A549 cells with ArBu and ArBu@PEG-PLA (Figure 3D). To gain better insight into ArBu@PEG-PLA-induced apoptosis in A549 cells, we measured the level of Bcl-2 and Bax in mitochondrial apoptosis signaling. The results showed that ArBu@PEG-PLA micelles significantly reduced the level of Bcl-2 and thus the anti-apoptotic Bcl-2/Bax ratio. Moreover, it has been reported that cytochromes can upregulate the expression of caspase3 and caspase 9 in the mitochondrial apoptosis pathway. Compared to free ArBu, the results showed that the expression of apoptosis-related proteins such as cytochrome C, caspase 9, and caspase 3 was significantly increased in A549 cells treated with ArBu@PEG-PLA. These results further confirmed that apoptosis is related to the mitochondrial pathway.

### 3.9. Acute toxicities of ArBu@PEG-PLA and ArBu

Acute toxicity tests of free ArBu and ArBu@PEG-PLA micelles were performed in KM mice prior to *in vivo* anti-tumor evaluation. Based on predetermined LD0 and LD100 values, different concentrations of free ArBu and ArBu@PEG-PLA micelles were administered in a single injection. Toxic reactions such as paralysis, muscle spasms, tachypnea, and arrhythmias were observed in all groups. All deaths occurred within 4 h of drug administration. The LD50 and 95% confidence limits of free ArBu were 15.601 and 12.931 − 18.635 mg/kg, respectively. The corresponding LD50 of ArBu@PEG-PLA micelles was 35.551 mg/kg, with 95% confidence limits of 33.998 − 38.103 mg/kg. The LD50 of ArBu@PEG-PLA micelles was 2.3-fold higher than that of free ArBu, demonstrating that the toxicity of ArBu was greatly improved by ArBu@PEG-PLA micelle encapsulation; thus, the safety is greatly improved.

No mice died after injection with 10 mg/kg free ArBu and 10 mg/kg ArBu@PEG-PLA micelles after 10 days of observation. Consequently, 5 or 10 mg/kg of free ArBu or ArBu@PEG-PLA micelles was chosen as the appropriately high and low doses for the *in vivo* anti-tumor evaluation.

### 3.10. In vivo tumor inhibition

**Figure 4.**
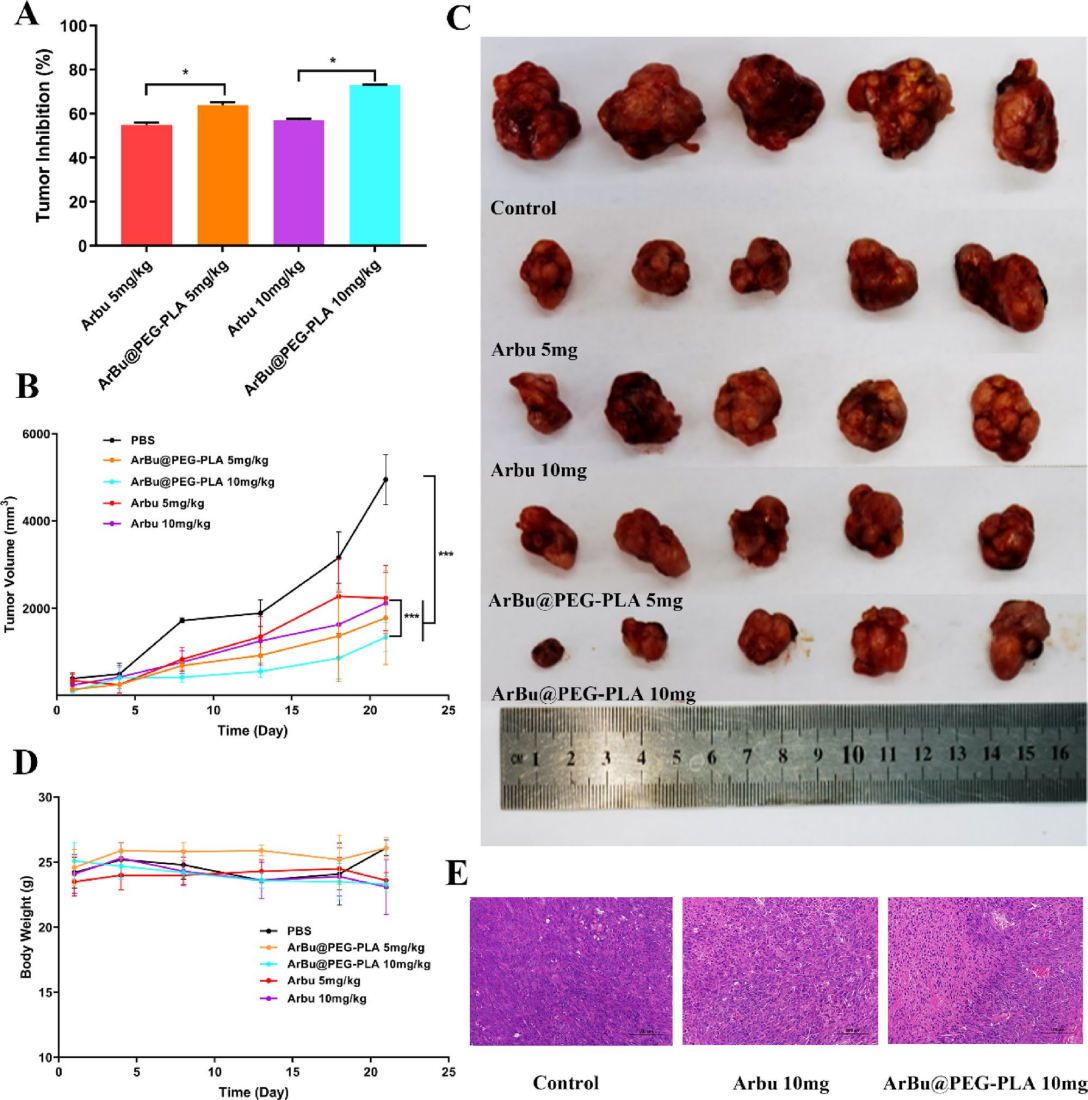
(A) The body weight of mice in various groups. (B) The tumor volume of A459 tumor-bearing mice after different treatments during 21 days, ***p < .001. (C) Photographs of excised tumors on 21th day. (D) H&E staining of tumors after different treatments. Scale bar, 100 μm.

The therapeutic effect of free ArBu and ArBu@PEG-PLA micelles was further assessed in A549 tumor-bearing mice. The A549 tumor-bearing mice were randomly divided into four groups and treated with (1) PBS, (2) free ArBu 5 mg/kg, (3) ArBu@PEG-PLA micelles 5 mg/kg, and (4) ArBu@PEG-PLA micelles 10 mg/kg. The anti-tumor therapeutic result is shown in Figure 4, and the tumor growth of the mice was monitored after different treatments. There were no obvious abnormalities in feeding, appearance, or activity of the mice during the subsequent 21 days. The tumor volume and tumor suppression rate of each experimental group are shown in Figure 4. All three ArBu formulations remarkably inhibited tumor growth. The inhibition rates at day 21 post treatment for the ArBu injection and ArBu@PEG-PLA micelle groups were 57.1% and 72.9%, respectively (Figure 4A). The mice tumor volumes were monitored during the study to evaluate the systemic anti-tumor effect of the formulations (Figure 4B). The tumor volume increased rapidly in the control group, but slow growth was observed in both the free ArBu and ArBu@PEG-PLA micelle groups. The total tumor size and growth rate of the ArBu@PEG-PLA micelle group were significantly lower than those of the free ArBu group (Figure 4C). The tumor-inhibition rate was positively correlated with the dose of ArBu. Histological analysis of the tumor tissue showed that the structure of the tumor tissue was abnormal after treatment with ArBu@PEG-PLA micelles in A549 tumor-bearing mice: most tumor cells showed obvious nuclear damage and died (Figure 4E), suggesting that ArBu@PEG-PLA micelles had enhanced anti-tumor effects. At the same dose of ArBu, small ArBu@PEG-PLA micelles could accumulate in the tumor site through the EPR effect due to the long-cycling characteristics of PEG-based systems. The ArBu was gradually released upon degradation of PLA, thus increasing the amount of drug at the tumor site. Therefore, the anti-tumor effect of micelles is more obvious than that of the free drug group, which is consistent with the *in vitro* studies.

**Figure 5.**
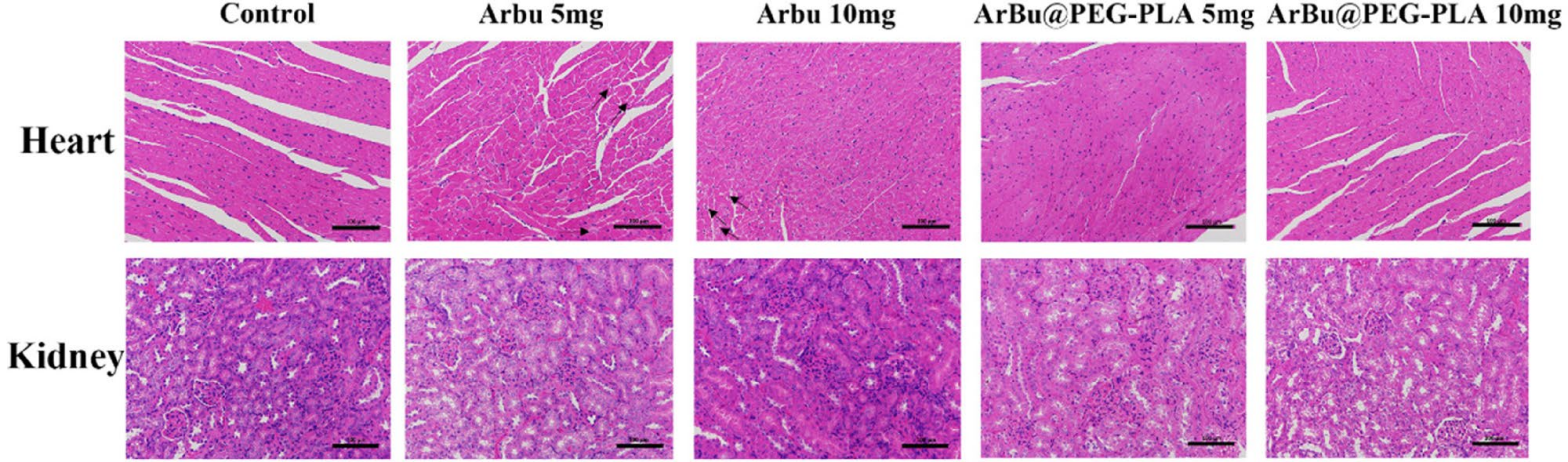
H&E staining of heart and kidney after different treatments. Scale bar, 100 μm.

We further investigated the biocompatibility of ArBu@PEG-PLA micelles: there was a negligible change in body weight during the 21 days of tumor therapy (Figure 4D). ArBu is known to have cardiotoxicity, and drug metabolism mainly involves the kidneys; thus, we focused here on lesions in these two major organs. These same models had histological analysis of heart and kidney to evaluate the systematic toxicity of different ArBu@PEG-PLA micelles in vivo by hematoxylin and eosin (HE) staining. Figure 5 shows the pathological analysis indicating that the cardiac muscles in tumor-bearing mice treated with free ArBu had extensive cytoplasmic vacuolization, but almost no vacuolization was observed in ArBu@PEG-PLA micelles. Thus, the low toxicity and safety of micelles were further confirmed. There was no obvious pathological damage to the kidneys in H&E staining, suggesting that the ArBu@PEG-PLA micelles were biocompatible and had low side effects during *in vivo* treatment.

## 4. Conclusion

We prepared ArBu-loaded polymeric micelles with a clear structure, uniform particle size, high drug loading capacity, good release ability, and high stability. All micelles had a high drug loading content (7.3–7.5%) and drug encapsulation efficiency (80.3–82.5%). The size was 71–74 nm, with a PDI of 0.114 even at 5 days. The ArBu@PEG-PLA micelles could also enhance tumor accumulation via the EPR effect. Compared to free ArBu, the polymer micelles could enhance internalization and induce apoptosis of A549 cells via a mitochondrial apoptosis pathway. The efficacy of the micelles was also confirmed via animal models: the tumor-inhibition rate of ArBu@PEG-PLA micelles was 72.9%, which is 1.28-fold higher than that of free ArBu, thus showing good tumor treatment effects. The ArBu@PEG-PLA micelles thus significantly improved the bioavailability in mice and showed low toxicity and high safety. Therefore, the ArBu-loaded polymeric micelles exhibited high potential for adjuvant cancer therapy and clinical translation.
